# miR-21 is upregulated, promoting fibrosis and blocking G2/M in irradiated rat cardiac fibroblasts

**DOI:** 10.7717/peerj.10502

**Published:** 2020-12-10

**Authors:** Huan Guo, Xinke Zhao, Haixiang Su, Chengxu Ma, Kai Liu, Shanshan Kong, Kedan Liu, Haining Li, Juan Chang, Tao Wang, Hongyun Guo, Huiping Wei, Zhaoyuan Fu, Xinfang Lv, Yingdong Li

**Affiliations:** 1 School of Basic Medical Sciences, Lan Zhou University, Lan Zhou, Gan Su, China; 2 Gansu University of Chinese Medicine, Lan Zhou, Gan Su, China; 3 Gansu Provincial Academic Institute for Medical Sciences, Gansu Provincial Cancer Hospital, Lan Zhou, Gan Su, China; 4 Department of Interventional Section, Affiliated Hospital of Gansu University of Chinese Medicine, Lan Zhou, Gan Su, China; 5 Chinese Academy of Medical Sciences, Fuwai Hospital, Bei Jing, China; 6 Department of Endocrinology, The First Hospital of Lanzhou University, Lanzhou, Gansu, China

**Keywords:** Heart, CFs, X-ray, WGCNA and DEG, Fibrosis, Cell cycle

## Abstract

**Background:**

Radiation exposure of the thorax is associated with a greatly increased risk of cardiac morbidity and mortality even after several decades of advancement in the field. Although many studies have demonstrated the damaging influence of ionizing radiation on cardiac fibroblast (CF) structure and function, myocardial fibrosis, the molecular mechanism behind this damage is not well understood. miR-21, a small microRNA, promotes the activation of CFs, leading to cardiac fibrosis. miR-21 is overexpressed after irradiation; however, the relationship between increased miR-21 and myocardial fibrosis after irradiation is unclear. This study was conducted to investigate gene expression after radiation-induced CF damage and the role of miR-21 in this process in rats.

**Methods:**

We sequenced irradiated rat CFs and performed weighted correlation network analysis (WGCNA) combined with differentially expressed gene (DEG) analysis to observe the effect on the expression profile of CF genes after radiation.

**Results:**

DEG analysis showed that the degree of gene changes increased with the radiation dose. WGCNA revealed three module eigengenes (MEs) associated with 8.5-Gy-radiation—the Yellow, Brown, Blue modules. The three module eigengenes were related to apoptosis, G2/M phase, and cell death and S phase, respectively. By blocking with the cardiac fibrosis miRNA miR-21, we found that miR-21 was associated with G2/M blockade in the cell cycle and was mainly involved in regulating extracellular matrix-related genes, including *Grem1*, *Clu*, *Gdf15*, *Ccl7*, and *Cxcl1*. Stem-loop quantitative real-time PCR was performed to verify the expression of these genes. Five genes showed higher expression after 8.5 Gy-radiation in CFs. The target genes of miR-21 predicted online were *Gdf15* and *Rsad2*, which showed much higher expression after treatment with antagomir-miR-21 in 8.5-Gy-irradiated CFs. Thus, miR-21 may play the role of fibrosis and G2/M blockade in regulating *Grem1*, *Clu*, *Gdf15*, *Ccl7*, *Cxcl1*, and *Rsad2* post-irradiation.

## Introduction

Clinical studies have demonstrated that heart disease-related mortality risk is increased by radiation therapy of cancer (Hancock, Tucker & Hoppe, 1993; Van Leeuwen & Ng, 2016). Although currently used regimens lead to lower cardiovascular toxicity compared to traditional regimens (Rademaker et al., 2008), cardiotoxicity may still occur, leading to radiation-induced heart disease (RIHD) (Boero et al., 2016). Overall changes in the gene expression profile may be one of the mechanisms of RIHD because ionizing radiation of tissues and organs can damage key macromolecules such as DNA, proteins, and alter the gene expression profiles of heart cells (Freeman et al., 2014). However, few studies have evaluated RIHD in detail at the molecular level.

miR-21 plays an important role in heart disease (Kura et al., 2020), and was known as an active gene in cardiac fibroblast (CF) and fibrosis (Thum et al., 2008). In primary cultured CFs, inhibition of miR-21 expression reduces collagen and extracellular matrix molecule genes expression, which are highly expressed during cardiac fibrosis (Thum et al., 2008). Inhibition of miR-21 can alleviated cardiac fibrosis by NF-κB/ miR-21 / SMAD7 pathway (Li et al., 2020), miR-21/ Jagged1 pathway (Zhou et al., 2018), miR-21/ Sprouty 1 pathway (Adam et al., 2012). Elevation of miR-21 not only promotes the expression of extracellular matrix genes, but also promotes proliferation and the transition of rat CFs into myofibroblasts (Zhou et al., 2018). These effects are involved in radioactive myocardial pathology (Wang et al., 2019).

We have shown that miR-21 expression in the rat heart is increased after radiation exposure (Ma et al., 2019), resulting in various pathological changes in the heart (Ma, Zhao & Li, 2019). CF account for 85% of heart cells (Moore-Morris et al., 2014), perform important functions, and are more sensitive to ionizing radiation than cardiomyocytes. However, how the increased miR-21 in CFs after radiation affects gene changes and participates in the development of radioactive heart disease is unclear. In the present study, we combined the weighted correlation network analysis (WGCNA) (Langfelder & Horvath, 2008) method with DEG analysis to examine changes in the gene expression profiles of primary CFs after irradiation, particularly following interference with miR-21 expression.

## Materials & Methods

### Experiment design

We performed WGCNA and DEG analysis of gene expression in X-ray-irradiated CFs. Because the degree of influence of irradiation on cells varies with the X-ray dose (Zhao et al., 2019), we used two doses: 1 Gy and 8.5 Gy. The cells were divided into a control group (un-irradiated, 0 Gy), 1 Gy group, and 8.5 Gy group. Additionally, to investigate which genes were regulated by miR-21 after radiation, interference of miR-21 in CFs was performed. Groups were defined as the anti-miR-21 group, anti-miR-21+1-Gy group, and anti-miR-21+8.5-Gy group. These six groups of cells were sequenced.

Each group was subjected to WGCNA and DEG analysis separately. The identified DEGs were compared with the module eigengenes (MEs) from WGCNA, and consensus genes from the MEs with DEGs (—log2FC— ≥ 1, adjusted *p*-value ≤ 0.05) were selected. Each group of consensus genes is referred to as a cluster; forinstance, in a consensus process, the same gene between DEGs in the 8.5-Gy group compared to 0-Gy group and in the brown module was denoted as the 8.5 vs 0-Gy cluster (Supplemental Information 1). Gene Ontology (GO) and Kyoto Encyclopedia of Genes and Genomes (KEGG) were used to analyze the functions of these genes.

**Figure 1:**
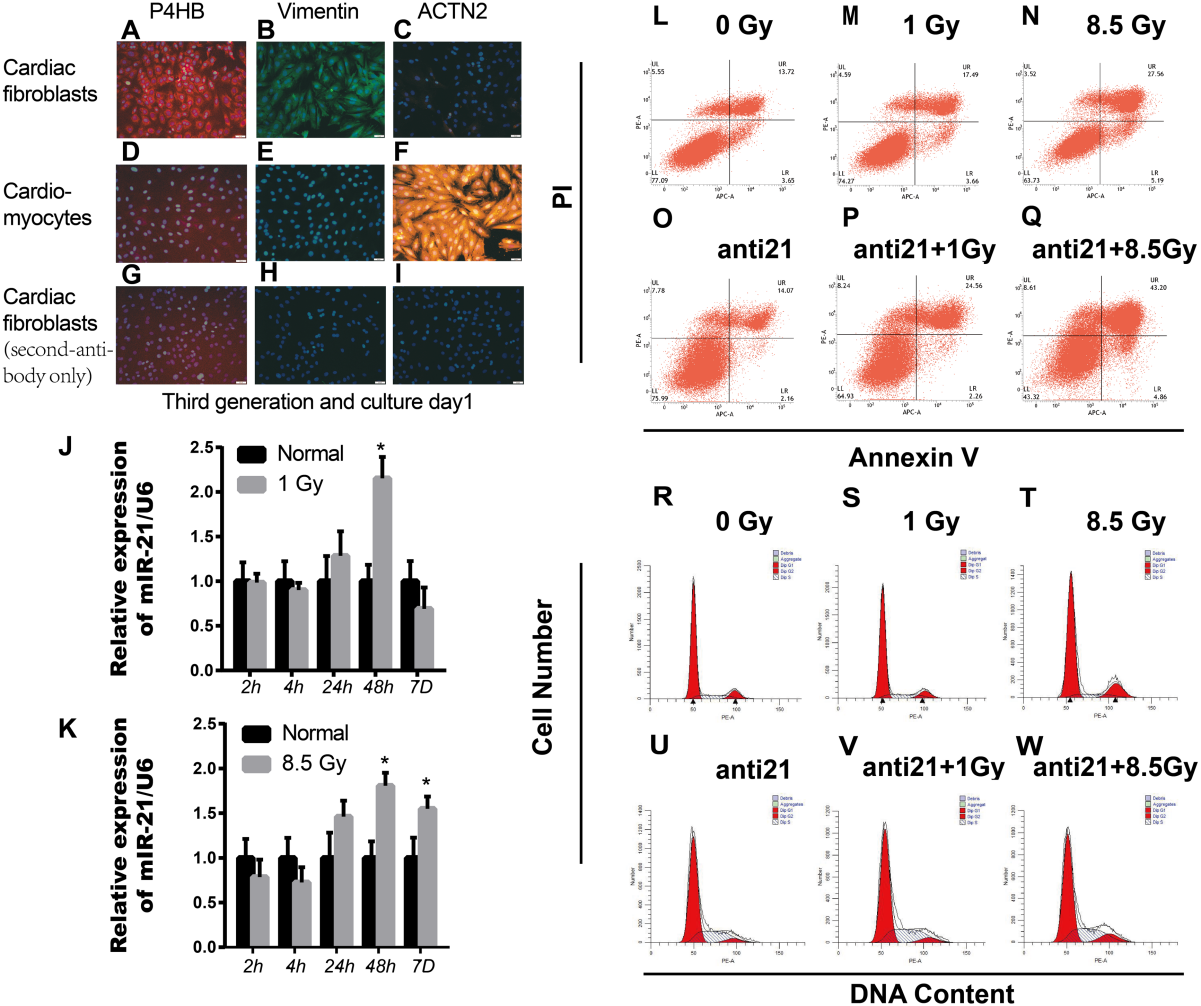
Effect of interference with miR-21 after radiation. (A–I) Cell fractions from rat hearts stained with DAPI and antibodies against prolyl 4-hydroxylase (P4HB), Vimentin and ACTN2. Scale bar, 20 µm. (J–K) miR-21 expression in primary cardiac fibroblasts (CFs) isolated from neonatal rat hearts after X-ray irradiation at different time points. ^∗^*p* < 0.05 vs controls. Data are presented as mean ±  S.E. Statistics: Two-tailed Student’s *t*-test. The experiment was repeated three times. (L–Q) Percentage apoptotic CFs after treatment with miR-21 antagonists (anti-miR-21) and irradiation (1Gy or 8.5Gy). (R–W) Cell cycle of CFs after treatment with miR-21 antagonists and irradiated with X-ray (1Gy or 8.5Gy).

Furthermore, the role of miR-21 after radiation was investigated; we chose new identified consensus genes (new-pick genes) in the target cluster showing significant down-regulation, and the expression level was attenuated in the anti-21+X-ray cluster according to log2FC (X-ray cluster)-log2FC (anti-miR21+X-ray cluster) >1 (Supplemental Information 1).

### Culture and isolation of cardiac fibroblasts (CFs) and cardiomyocytes (CMs)

Healthy new born Wistar rats (days 0–2) purchased from Laboratory animal Center of Gansu University of Chinese Medicine. Parent rats were raised in the Laboratory center in a comfortable room with a 12 h dark/12 h light cycle and free access to food and water. Every sacrificed newborn rat comes from the same parents above. Cultivation of CFs and cardiomyocytes (CM) from new-born rats were cultured as described previously with some modifications (Thum et al., 2008). Briefly, rats were sacrificed after isoflurane (2%) inhalation and cervical dislocation. Primary cardiac fibroblasts and cardiomyocytes were separated from 4-6 mice cardiac tissue each time. The collected cells were plated in MEM-F12 containing vitamin B12, NaHCO_3_, and 10% fetal calf serum. The cultures contained mostly primary cardiac fibroblasts, as >95% of cells were stained with antibodies for fibroblast-specific antigen prolyl-4-hydroxylase (P4HB, ab137110, Abcam, Cambridge, UK) and Anti-Vimentin antibody (ab92547, Abcam, Cambridge, UK), and >95% of cells were negative for the cardiomyocyte-specific marker *α*2-actinin (ACTN2, clone EA-53, Sigma) (Figs. 1A–1I). We cared for the animals and sacrificed the rats in strict accordance with animal welfare laws and regulations and animal welfare ethics requirements. This study was approved by the Ethical Committee (the certificate number: GZY2018-115, Date: 03-05-2018) of Gansu University of Chinese Medicine in Lan Zhou, China.

### RNA isolation and gene expression

Total RNA containing small RNA was extracted using the miRVanaTM RNA Isolation Kit AM1561 (Invitrogen, Carlsbad, CA, USA) according to the manufacturer’s instructions. RNAs were transcribed to cDNA using the SuperScript™ III Reverse Transcriptase kit (Invitrogen).Stem-loop quantitative real-time PCR (sqRT-PCR) assay was performed to validate the miRNA, Q-PCR was conducted to detect mRNA expression levels using the following conditions: 95 °C for 5 min, 35 cycles of 95 °C for 15 s, 60 °C for 30 or 60 s, and 72 °C for 20 s. The relative expression level of miRNA/mRNA was normalized to U6/glyceraldehyde 3-phosphate dehydrogenase and fold-change in the miRNA/mRNA level was calculated with the 2-^△△Ct^ method. Each sample was analyzed in triplicate. All primers were obtained from RiboBio (Guangzhou RiboBio Co., Guangzhou, China).

### Assay for transfection, apoptosis, and cell cycle

Rat CFs were transfected with antagomir of miR-21 (100nM, RiboBio) using HiPerFect Transfection Reagent (Cat No./ID:301705, Qiagen, Hilden, Germany). The percentage of CFs undergoing apoptosis was determined by staining with annexin V (Annexin V: FITC Apoptosis Detection Kit I, 556547, BD Biosciences, Franklin Lakes, NJ, USA) followed by fluorescence-activated cell sorting analysis. The cell cycle were evaluated by propidium iodide (PI) staining.

### Ionizing radiation treatment

Irradiation of cell cultures containing 1 ×10^6^ log phase cells was performed with a precision X-RAY irradiator (X-RAD 225, Precision X-ray, North Branford, CT, USA) at a dose rate of 2 Gy/min. Doses of 1 Gy and 8.5 Gy were administered at room temperature, and control cells were sham-irradiated.

### Illumina sequencing

Next-generation sequencing was performed with the Illumina NovaSeq6000 instrument (San Diego, CA, USA) at Beijing CapitalBio Technology Company (Beijing, China). Normalized gene expression values were calculated using the expected number of fragments per kilobase of transcript sequence per millions base pairs sequenced (FPKM) method (Mortazavi et al., 2008).

### WGCNA construction

**Figure 2:**
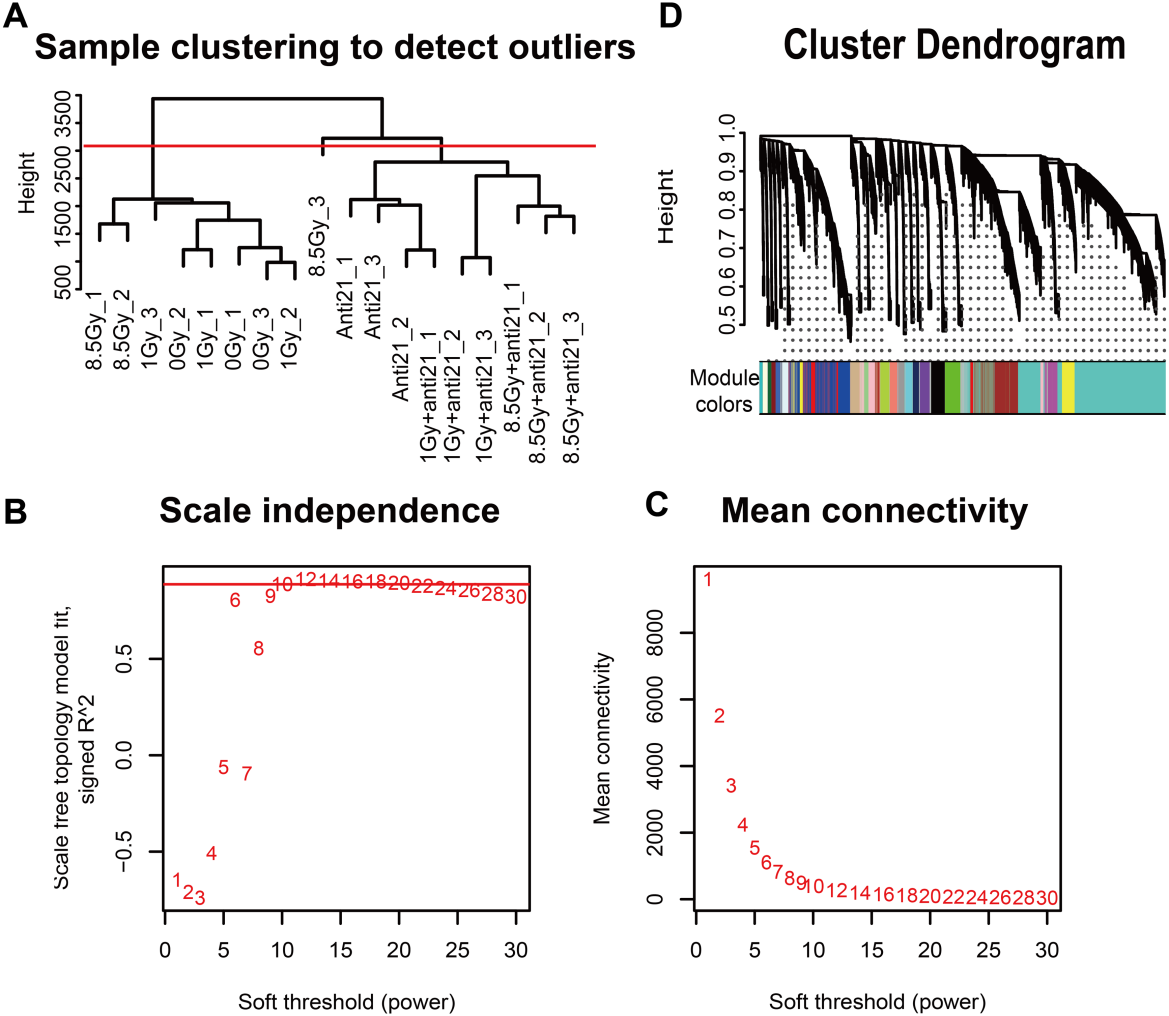
WGCNA of CFs in different groups. (A) Dendrogram depicting hierarchical clustering to detect outlier samples (8.5-Gy-3 is an outlier sample). (B–C) Scale independence and mean connectivity were used for soft threshold selection in WGCNA. (D) Hierarchical cluster trees showing co-expression modules identified by WGCNA.

Gene hierarchical cluster network was constructed using the WGCNA R package (Langfelder & Horvath, 2008) and visualized in a dendrogram. All samples were clustered, and outlier samples (cutHeight = 3000) were excluded from data analysis (Fig. 2A). A β-soft power threshold of 10 was selected to ensure that the network satisfied a scale-free topology (R^2^ > 0.9) based on the linear regression model fitting index obtained from the functions ’pickSoftThreshold’ operation (Figs. 2B–2C). Coexpression modules were detected using the function ‘blockwiseModules’ with default settings and modified parameters (power, 10; minModuleSize, 50; mergeCutHeight, 0.25). The grey modules indicate nonsense areas. Based on each module eigengene, correlation analysis was performed to identify modules that were significantly associated with the measured traits (apoptosis and cell cycle).

### Identification of differentially expressed genes

‘DESeq2’ package in R was used to identify DEGs (Love, Huber & Anders, 2014). To control the false discovery rate, *p*-values were adjusted. Values of —log2FC—≥ 1 and padj ≤ 0.05 were considered as different.

### Bio-function enrichment analysis

The Database for Annotation, Visualization and Integrated Discovery (DAVID) (DennisJr et al., 2003) was used to perform GO function and KEGG pathway enrichment analysis. The GO terms of biological processes (BP), cellular components (CC), and molecular functions (MF) were assessed. A *p*-value <0.05 was considered to indicate a significant difference for the GO terms and KEGG pathways.

### UpSet veen analysis

An UpSet veen map was constructed using online omicshare soft ( http://www.omicshare.com/tools/Home/Soft/getsoft).

### Identification of protein–protein interaction networks

The online Search Tool for the Retrieval of Interacting Genes (STRING database, Version11.0; http://string-db.org/) was used to build a protein-protein interaction (PPI) network (Szklarczyk et al., 2019) (threshold of > 0.4).

### Statistical analysis

All experimental data statistical analyses were carried out using GraphPad Prism software (version 7.0; GraphPad, Inc., La Jolla, CA, USA), and the data are expressed as the mean ± standard error of mean. Statistical differences between two groups were calculated by two-tailed Student’s *t*-test, and *p* < 0.05 was considered significant.

## Results

### Upregulation of miR-21 in irradiated CFs

The sqRT-PCR results revealed upregulation of miR-21 in irradiated CFs, which persistently increased up to 7 days after 8.5 Gy irradiation (Fig. 1K); however, miR-21 expression did not increase with increasing radiation intensity. In addition, there were more apoptotic cells after irradiation, which increased with irradiation intensity, compared to non-irradiated control cells. Importantly, many more apoptotic cells were observed after interference of miR-21 with irradiation (Figs. 1L–1Q). Cell cycle analysis showed that X-rays can stall cells in the G2/M phase; however, this effect was strongly attenuated by treatment with the miR-21 inhibitor (Figs. 1R–1W). These results indicate that miR-21 has an important function in CFs.

### Data processing and construction of weighted coexpression network and identification of key modules

To explore the co-expression patterns of CFs genes after irradiation, RNA-seq FPKM data from 18 samples were evaluated with the WGCNA package (Langfelder & Horvath, 2008) (Fig. 2). Results from the 8.5-Gy-3 sample were outliers and thus were excluded (Fig. 2A). We found 24 different MEs according to their degree of connectivity (Fig. 2D). The number of genes contained in each module is shown in Supplemental Information 2.

**Figure 3:**
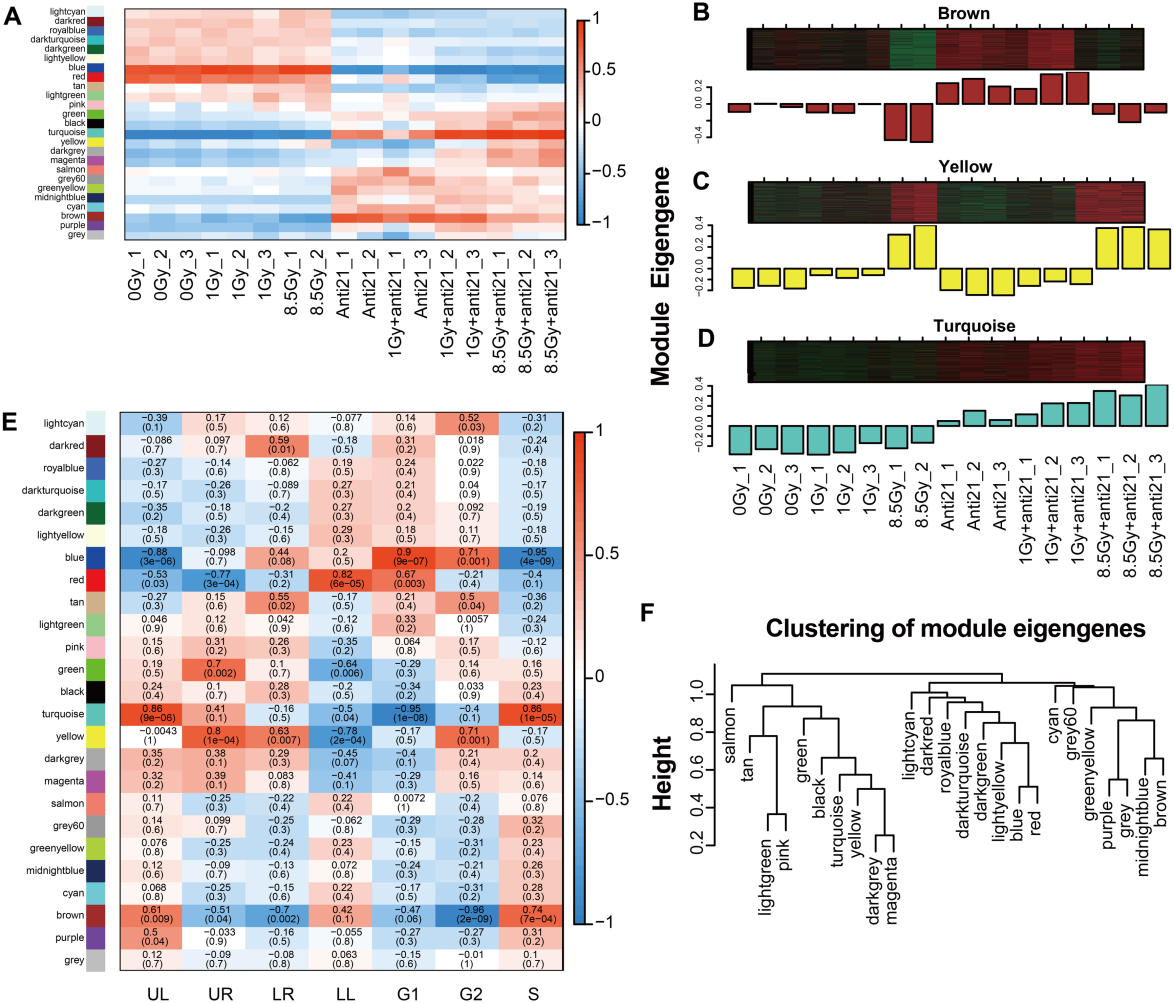
WGCNA analysize for module-trait relationships and Module expression pattern. Matrix showing module-trait relationships (MTRs) for CFs of different groups (A) and for (E) cell apoptosis and cell cycle. (A) Each row corresponds to a module. Each column corresponds to a sample result. (E) Each row corresponds to a module. Each column corresponds to a phenotype result. The MTRs are colored based on their correlation: red indicates a strong positive correlation and blue indicates a strong negative correlation. Module expression pattern (B–D) and clustering of module eigengenes (F). (B) Heat map represents the expression of genes where each row represents a gene and each column represents a sample. The red color in the heat map represents up-regulated genes, whereas the green color represents the down-regulated gene; the bar charts represents the eigengene profiles; the color of the bar chart represents the color of related modules. (D) Dendrogram depicting hierarchical clustering of module eigengenes. The relationships among the modules are shown in the dendrogram. An increased predictive power of the main effects on module expression in the dendrogram (closer clustering).

**Figure 4:**
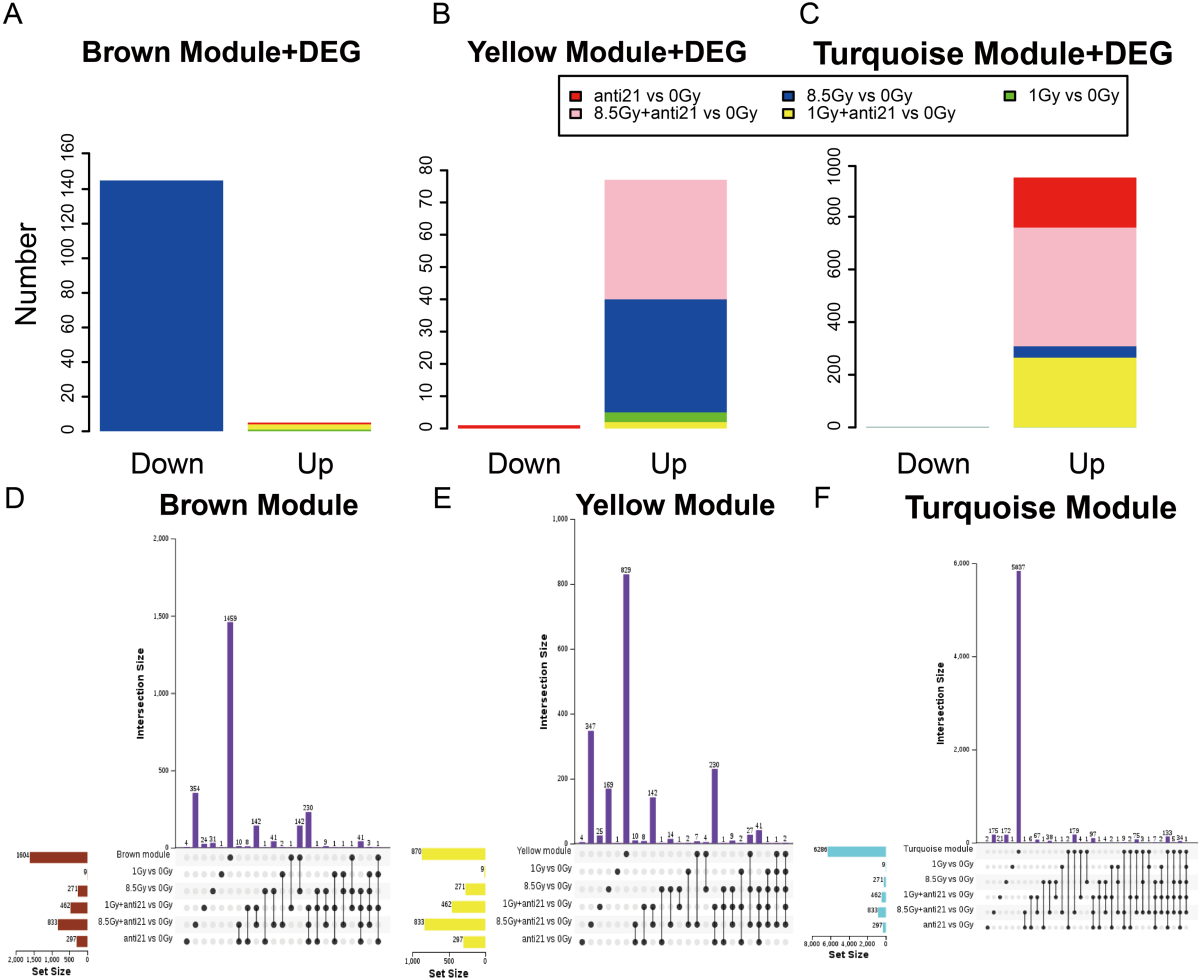
Trend of Consensus of DEG with MEs. (A–C) Bar chart of consensus genes from DEGs with three modules. (D–F) UpSet veen map showing the number of expressed genes detected in brown, yellow, and turquoise module consensus with DEGs among different groups. Black dots denote the number of unique genes in target module or DEGs groups; point-line connections represent the number of genes shared by two or more DEGs groups or target module.

To analyze the expression of ME in each group, module genes of 17 samples were analyzed. We drew heat maps of gene expression (Fig. 3A). In the Brown module, gene expression was down-regulated after irradiation and severely down-regulated in the 8.5-Gy group but increased in the anti-21 and anti-21+1-Gy groups. In the 8.5-Gy+anti21 group, gene expression was down-regulated compared to in the anti-21 group but up-regulated compared to in the 8.5-Gy group. These results indicate that the Brown module has a central module relationship with miR-21. The Yellow and Turquoise modules showed the opposite gene expression pattern as the Brown module; after irradiation, gene up-regulation in the Turquoise module was intensified after interference with miR-21 (Fig. 3A, Figs. 4A–4C).

### Features (apoptosis and cell cycle) relationship with modules and identification of key modules

To clarify the relationship between apoptosis and cell cycle with the modules, 24 different MEs were analyzed and a heat map was generated (Figs. 2D, 3E). Three modules showed notable results (Figs. 3B–3D). Brown module showed a significant negative correlation with G2/M phase, with a correlation index of −0.96 (*p* = 2 ×10^−9^); Yellow module, associated with late apoptosis, exhibited a correlation index of 0.80 (*p* = 1 ×10^−4^); and Turquoise module which is related to cell death and S phase showed a correlation index of 0.86 (*p* = 9 ×10^−6^; *p* = 1 ×10^−5^).

### Correlation between modules and identification of key modules

Among the 24 different MEs, interaction associations were analyzed, and a tree map was constructed (Fig. 3F). The results showed that modules were independent of each other.

### DEGs and consensus DEGs in target modules

To identify the key genes in MEs, we performed DEG analysis and evaluated consensus genes showing differential expression within different modules. DEGs (Supplemental Information 3) in the 1-Gy, 8.5-Gy, 1-Gy+anti21, and 8.5-Gy+anti21 (vs 0-Gy) groups were used to identify genes within the three WGCNA modules (Brown, Yellow, and Turquoise) (Figs. 4A–4C). After each module and DEGs were screened and filtered, the consensus genes were used to draw an UpSet veen map (Figs. 4D–4F). Subsequently, we selected 1-Gy vs 0-Gy cluster genes and 8.5-Gy vs 0-Gy cluster genes from the three modules for GO and KEGG analysis.

### GO functional analysis of X-ray irradiated CFs

**Figure 5:**
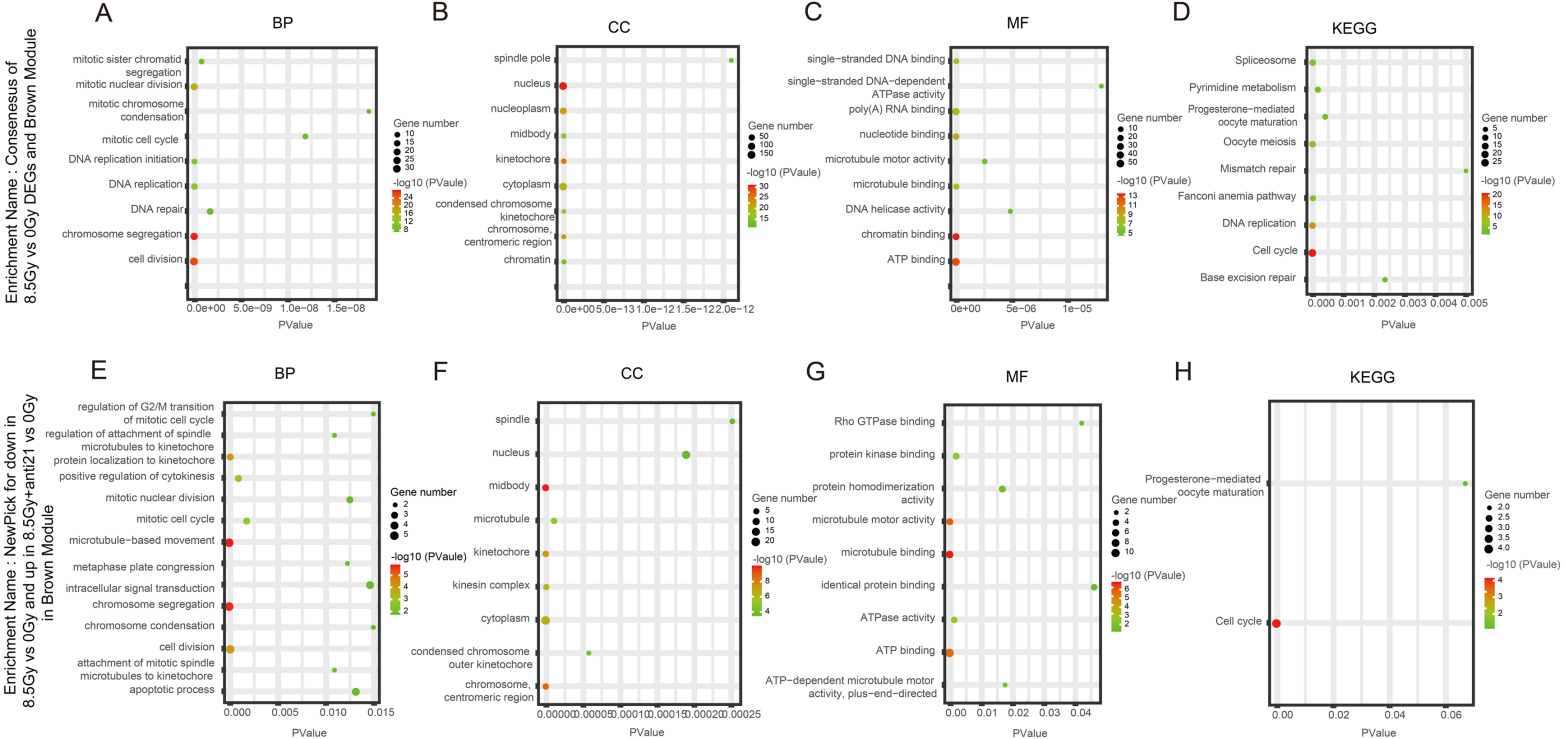
GO and KEGG pathway enrichment analysis of DEGs and Brown module. (A–D) GO and KEGG pathway enrichment analysis of cluster of 8.5-Gy vs 0-Gy DEGs and Brown module. (E–H) New-pick genes for down-regulation8.5-Gy vs 0-Gy cluster and up-regulation.

**Figure 6:**
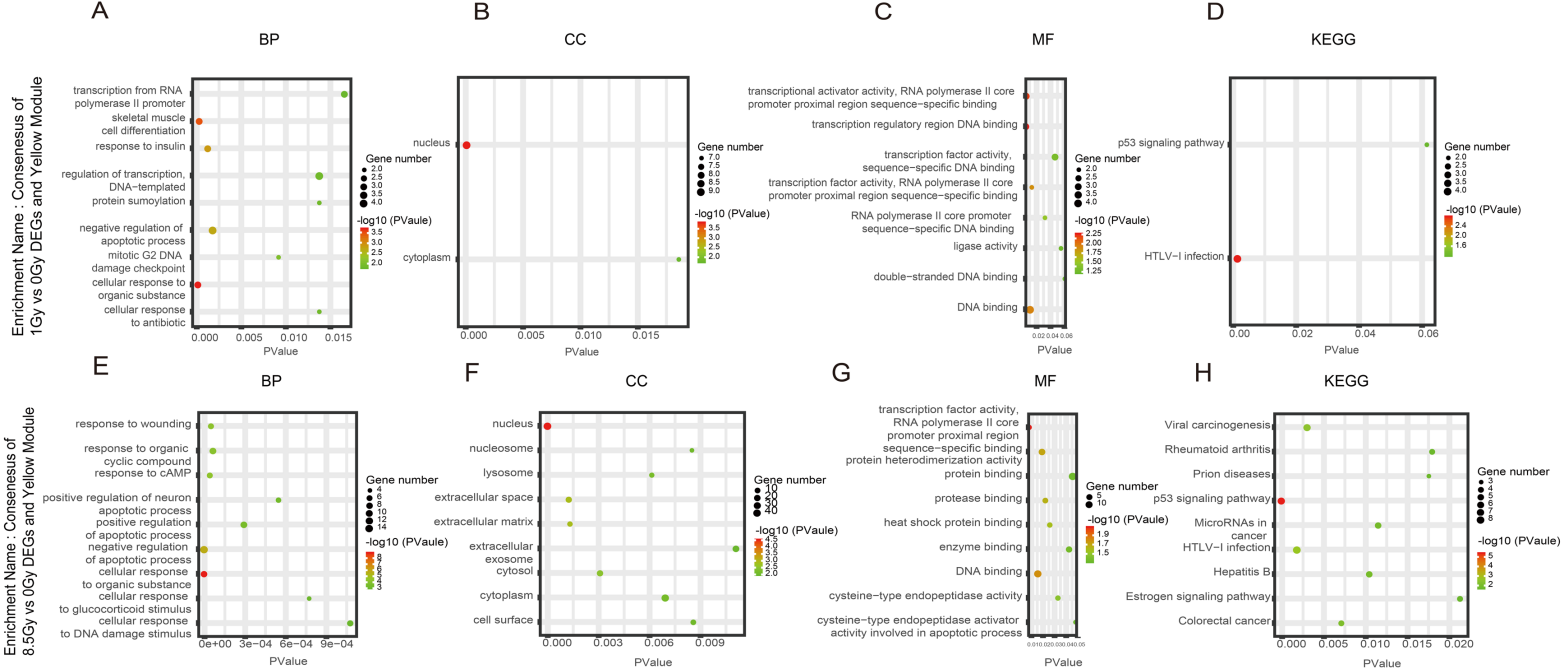
GO and KEGG pathway enrichment analysis of DEGs and Yrown module. (A–D) GO and KEGG pathway enrichment analysis of consensus of 1-Gy vs 0-Gy DEGs and Yellow module. (E–H) GO and KEGG pathway enrichment analysis of consensus of 8.5-Gy vs 0-Gy DEGs and Yellow module. From left to right, GO biological process (first), cellular component (second), molecular function (third), and KEGG pathway (fourth). GO, Gene Ontology; KEGG, Kyoto Encyclopedia of Genes and Genomes; DEGs, differentially expressed genes.

**Figure 7:**
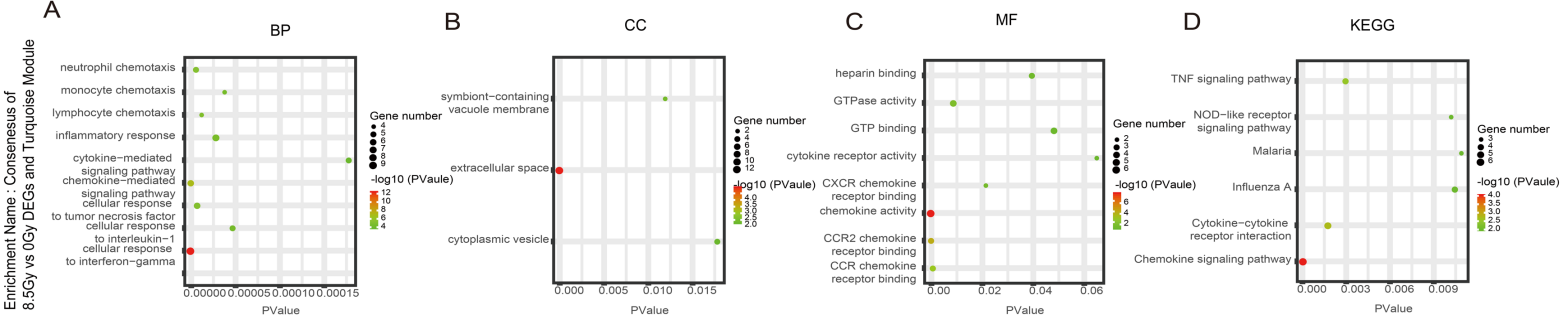
GO and KEGG pathway enrichment analysis of DEGs and Turquoise module. (A–D) GO and KEGG pathway enrichment analysis of consensus of 8.5-Gy vs 0-Gy DEGs and Turquoise module. From left to right, GO biological process (first), cellular component (second), molecular function (third), and KEGG pathway (fourth). GO, Gene Ontology; KEGG, Kyoto Encyclopedia of Genes and Genomes; DEGs, differentially expressed genes.

In the consensus study, we performed GO enrichment analysis of the consensus genes following evaluation with the DAVID web-based search tool. The enrichment is shown in Table 1 and (Figs. 5–7).

**Table 1:** GO and KEGG pathway enrichment analysis.

Module	Cluster	GO Term enrichment	KEGG enrichment
Brown	8.5Gy vs 0Gy	BP: cell division with DNA replication	Cell cycle, DNA replication, spliceosome, and DNA repair
		CC: nucleus, nucleoplasm, kinetochore, cytoplasm	
		MF: biomacromolecule binding	
Yellow	1Gy vs 0Gy	BP: organic substance, skeletal muscle cell differentiation, response to insulin, negative regulation of apoptotic process	HTLV-1 infection
		CC: nucleus, but also the extracellular space	
		MF: transcriptional activity, RNA polymerase II core promoter proximal region sequence-specific binding, transcription regulatory region DNA binding	
Yellow	8.5Gy vs 0Gy	BP: cellular response to organic substance, negative regulation of apoptotic process, muscle contraction	P53 signalling pathway, HTLV-1 infection, viral carcinogenesis, and MAPK signalling pathway
		CC: the extracellular matrix	
		MF: transcriptional activity, RNA polymerase II core promoter proximal region sequence-specific binding, protein heterodimerization activity	
Turquoise	8.5Gy vs 0Gy	BP: cellular response to interferon-gamma, chemokine-mediated signalling pathway	Chemokine signalling pathway
		CC: extracellular space	
		MF: chemokine activity, CCR2 chemokine receptor binding	
Brown	(anti-21+8.5Gy vs 0Gy) vs (down in 8.5Gy vs 0Gy) (log>1)	BP: microtubule-based movement, chromosome segregation, cell division, in regulation of the G2/M transition in the mitotic cell cycle	Cell cycle
		CC: midbody, chromosome, centromeric region, cytoplasm.	
		MF: microtubule binding, protein kinase binding, microtubule motor activity, ATP binding.	

In the Brown module, among BP, the cluster of 8.5Gy vs 0Gy was significantly associated with cell division, including chromosome segregation and mitotic nuclear division, and with DNA replication, including DNA replication and DNA replication initiation (Fig. 5A). In CC enrichment analysis, the terms nucleus, nucleoplasm, kinetochore, and cytoplasm were enriched (Fig. 5B). In MF analysis, the 8.5Gy vs 0Gy clusters were significantly associated with biomacromolecule binding, including chromatin binding, ATP binding, nucleotide binding, poly(A) RNA binding, and so on (Fig. 5C). However, in the 1Gy vs 0Gy clusters, only the nuclear envelope integral membrane protein 1 gene was up-regulated, and thus GO and KEGG analysis were not performed.

For genes in the Yellow module, among the BP, the 1Gy vs 0Gy cluster was significantly associated with the cellular response to organic substance, skeletal muscle cell differentiation, response to insulin, and negative regulation of apoptotic process (Fig. 6A). In the cluster of 8.5Gy vs 0Gy, the same results were observed as in the 1Gy vs 0Gy cluster, which were associated with cellular response to organic substance, negative regulation of apoptotic process, and mainly enriched in muscle contraction (Fig. 6E). In CC enrichment analysis, terms related to not only the nucleus but also the extracellular space were enriched in the 1Gy vs 0Gy cluster (Fig. 6B). The extracellular matrix was also enriched in the 8.5Gy vs 0Gy cluster, which is closely related to cellular fibrosis (Fig. 6F). In MF analysis, the 1Gy vs 0Gy cluster was significantly associated with transcriptional activity, RNA polymerase II core promoter proximal region sequence-specific binding, and transcription regulatory region DNA binding (Fig. 6C), whereas the 8.5Gy vs 0Gy cluster was also significantly associated with transcriptional activity, RNA polymerase II core promoter proximal region sequence-specific binding, and protein heterodimerization activity (Fig. 6G).

For genes in the Turquoise module, among the BP, the 8.5Gy vs 0Gy cluster was mainly enriched in cellular response to interferon-gamma and chemokine-mediated signalling pathway (Fig. 7A). In CC enrichment analysis, terms related to the extracellular space were enriched in 8.5Gy vs 0Gy (Fig. 7B). In MF analysis, our results showed that 8.5Gy vs 0Gy were significantly associated with chemokine activity and CCR2 chemokine receptor binding (Fig. 7C). However, in the 1Gy vs 0Gy cluster, only the BTG anti-proliferation factor 2 gene was up-regulated and chromosome 14 open reading frame 132 (D430019H16Rik) was down-regulated.

### KEGG pathway analysis

KEGG pathway maps of biological functions were obtained from the DAVID web-based search tool (Huangda, Sherman & Lempicki, 2009). In WGCNA, the Yellow and Brown modules showed a greater correlation with apoptosis, cell cycle, and cellular fibrosis compared to the other modules. We performed KEGG analysis to explore differences in the pathways in each cluster, See Table 1 and (Figs. 5D, 6D, 6H, 7D).

In the Brown module, the 8.5Gy vs 0Gy cluster was mainly enriched in the cell cycle, DNA replication, spliceosome, and DNA repair (Fig. 5D). In the Yellow module, the 8.5Gy vs 0Gy cluster was significantly associated with the P53 signalling pathway, HTLV-1 infection, viral carcinogenesis, and MAPK signalling pathway (Fig. 6H), and the 1Gy vs 0Gy cluster was significantly associated with HTLV-1 infection (Fig. 6D). In the Turquoise module, the 8.5Gy vs 0Gy cluster was significantly associated with the chemokine signalling pathway (Fig. 7D).

### GO functional analysis and KEGG analysis of CFs after anti-miR-21 treatment and irradiation

We observed the response of cells to radiation after interfering with miR-21. As shown in Figs. 5E–5H in the Brown module, after 8.5 Gy irradiation (compared to 0 Gy), some genes were down-regulated; whereas, after 8.5Gy irradiation and interference miR-21 (compared to 0 Gy), only a slight decreased in the genes involved in the former group was observed. We selected these genes for GO and KEGG analysis (Table 1). BP analysis showed that functional genes were mainly concentrated in microtubule-based movement, chromosome segregation, and cell division, and were involved in regulation of the G2/M transition in the mitotic cell cycle, which agrees with our cell cycle results. CC analysis showed that functional genes were mainly centralized in the midbody, chromosome, centromeric region, and cytoplasm. MF analysis demonstrated that functional genes were mainly concentrated in microtubule binding, protein kinase binding, microtubule motor activity, and ATP binding. KEGG analysis clearly revealed that these genes were associated with the cell cycle.

### PPI network analysis

**Figure 8:**
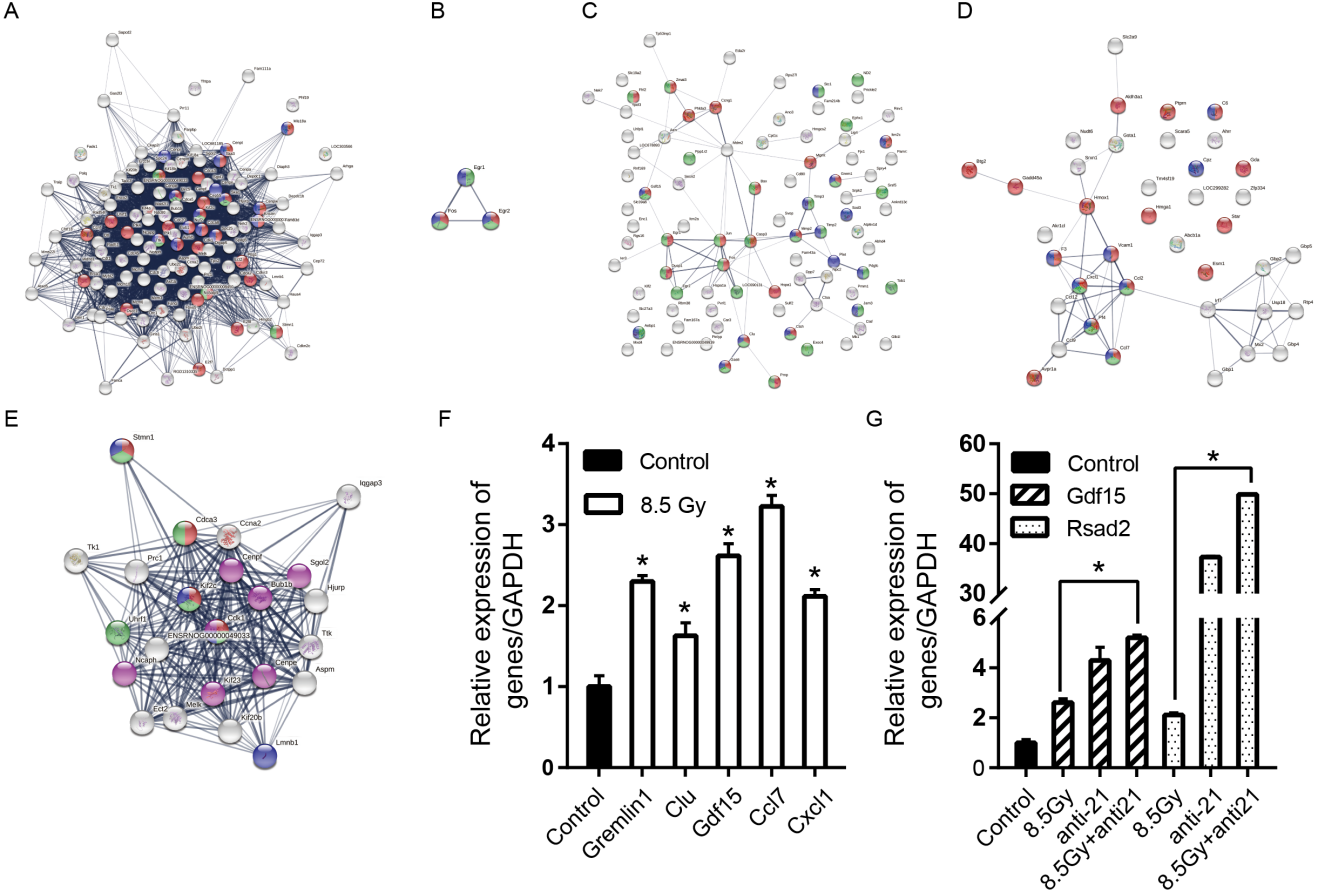
Consensus of DEG-WGCNA module network, PPI network and hub genes. (A) 8.5-Gy vs 0-Gy with Brown module (degree >20) network. Red cycle represent BP GO term (GO:0007049, cell cycle); green circles represent MF GO term (GO:0000775, chromosome centromeric region) and blue circles represent CC GO term (GO:0051301, cell division). (B) 1-Gy vs 0-Gy group with Yellow module (degree >2) network. Red cycle represent BP GO term (GO:0035914, skeletal muscle cell differentiation); green circles represent MF GO term (GO:0008134 transcription factor binding) and blue circles represent CC GO term (GO:0005634 nucleus). (C) 8.5-Gy vs 0-Gy with Yellow module (degree >2) network. Red cycle represents BP GO term (GO:0010941, regulation of cell death); green circles represent MF GO term (GO:0005515, protein binding) and blue circles represent CC GO term (GO:0005615, extracellular space). (D) 8.5-Gy vs 0-Gy with Turquoise module (degree >2) network. Red cycle represent BP GO term (GO:0048522, positive regulation of cellular proliferation); green circles represent MF GO term (GO:0008009, chemokine activity) and blue circles represent CC GO term (GO:0005615, extracellular space). (E) Cluster of 8.5-Gy vs 0-Gy group and 8.5-Gy+anti21 vs 0-Gy with Brown module network. Red circle represents BP GO term (GO:0051301, cell division); green circles represent MF GO term (GO:0007049, cell cycle) and blue circles represent CC GO term (GO:0099513, polymeric cytoskeletal fibre); purple circles represent reactome pathways (RNO-68886). (F) Relative expression of Grem1, Clu, Gdf15, Ccl7, and Cxcl1 after 8.5Gy irradiation of CFs. (G) Relative expression of Gdf15 and Rsad2 after 8.5Gy irradiation and anti-21 of CFs. PPI, protein-protein interaction; DEGs, differentially expressed genes. ∗*p* < 0.05 vs controls. Data are presented as mean ±  S.E. Statistics: Two-tailed Student’s *t*-test. The experiment was repeated three times.

To further investigate the function of the cluster genes at the protein level, we constructed a PPI network of the clusters (Figs. 8A–8E). Genes and GO terms of clusters of three modules; see Table 2 and Figs. 8A–8E. We further explored which genes and biological processes were being regulated by miR-21 elevation post-irradiation. Our study suggested that compared to the control (0 Gy), the Brown module gene was mainly down-regulated in irradiated cells, and there was a higher probability that the Brown module contained the target gene of miR-21 (Table 3).

**Table 2:** Names of three module genes of PPI network based on the STRING database.

Module	Degree	Cluster	GO term	Gene number	Genes
Brown	>20	8.5Gy vs 0Gy	BP (GO:0007049, cell cycle)	30	*Mis18a*, *Cenpt*, *Ska3*, *Kif18b*, *Cdca3*, *Birc5*, *Cep55*, *Ska1*, *Kif2c*, *Cenpw*, *Knstrn*, *Ccnf*, *Brca1*, *Uhrf1*, *Plk4*, *Plk1*, *Cdc20*, *Nuf2*, *Cdca8*, *Spc25*, *Aurkb*, *Cdk1*, *Pttg1*, *Cdkn3*, *Ns5atp9*, *Mcm6*, *E2f8*, *Stmn1*, *E2f7*, *Kifc1*
			MF (GO:0000775 chromosome centromeric region)	7	Kif18b, *Birc5*, *Ska1*, *Kif2c*, *Plk1*, *Kifc1*, *Stmn1*
			CC (GO:0051301 cell division)	15	*Mis18a*, *Cenpt*, *Cenpi*, *Birc5*, *Cenpn*, *Ska1*, *Cenpw*, *Knstrn*, *Kif2c*, *Nuf2*, *Bub1*, *Cdca8*, *Plk1*, *Aurkb*, *Spc25*
Yellow	>2	8.5Gy vs 0Gy	BP (GO:0010941, regulation of cell death)	8	*Ccng1*, *Gdf15*, *Egr1*, *Fos*, *Dusp1*, *Clu*, *Mgmt*, *Grem1*
			MF (GO:0005515, protein binding)	9	*Gdf15*, *Egr1*, *Fos*, *Dusp1*, *Egr2*, *Clu*, *Stc1*, *Ephx1*, *Grem1*
			CC (GO:0005615, extracellular space)	4	*Gdf15*, *Clu*, *Stc1*, *Grem1*
Turquoise	>2	8.5Gy vs 0Gy	BP (GO:0048522, positive regulation of cellular proliferation)	17	*Aldh3a1*, *Ptprn*, *C6*, *Gda*, *Star*, *Hmga1*, *Esm1*, *Gadd45a*, *Hmox1*, *F3*, *Vcam1*, *Cxcl1*, *Ccl2*, *Pf4*, *Ccl7*, *Avpr1a*, *Esm1*
			MF (GO:0008009, chemokine activity)	4	*Cxcl1*, *Ccl2*, *Pf4*, *Ccl7*
			CC (GO:0005615, extracellular space)	8	*C6*, *Cpz*, *F3*, *Vcam1*, *Cxcl1*, *Ccl2*, *Pf4*, *Ccl7*
Yellow	>2	1Gy vs 0Gy	BP (GO:0035914, skeletal muscle cell differentiation)	2	*Fos*, *Egr2*
			MF (GO:0008134, transcription factor binding)	3	*Fos*, *Egr2*, *Egr1*
			CC (GO:0005634, nucleus)	3	*Fos*, *Egr2*, *Egr1*

**Table 3:** Newly selected genes as potential targets of miR-21 in irradiation CFs.

Module	Cluster	GO Term/Function	Gene number	Genes
Brown	8.5Gy vs 0Gy consensus with 8.5+anti-21 vs 0Gy	BP (GO :0051301, cell division)	4	*Stmn1, Cdca3, Kif2c, and Cdk1*
		MF (GO :0007049, cell cycle)	5	*Uhrf1, Stmn1, Cdca3, Kif2c, Cdk1*
		CC (GO:0099513, polymeric cytoskeletal fibre)	4	*Lmnb1, Stmn1, Kif2c, Cdk1*
		KEGG:reactome pathways (RNO-68886) and mitotic M stage (mainly for G2/M period conversion function)	7	*Ncaph, Cdk1, Kif23, Cenpe, Cenpf, Bub1b, Sgol2*

### Experimental validations

For the preliminary verification of the fibrosis of CFs after irradiation, the expression levels of *Grem1*, *Clu*, *Gdf15*, *Ccl7*, and *Cxcl1* were determined. The Q-PCR results showed that the mRNA expression levels of *Grem1*, *Clu*, *Gdf15*, *Ccl7*, and *Cxcl1* were significantly increased in the 8.5-Gy group in contrast to the controls (0 Gy) (Fig. 8F), showing 2.300 ± 0.073-, 1.630 ± 0.159-, 2.616 ± 0.149-, 3.225 ± 0.138-, and 2.115 ± 0.085-fold higher expression, respectively. We also validated the genes predicted online (Gdf15, Rsad2) as miR-21 targets (Fig. 8G). The results showed that the expression of these two genes was increased by 2.616 ± 0.149- and 2.115 ± 0.085-fold after irradiation, and increased by 5.207 ± 0.111- and 49.853 ± 0.058-fold after interference miR-21 and following radiation (*P* < 0.05).

## Discussion

Studies have shown that a broad range of cardiomyopathy, myocardial fibrosis disorders such as structure, phenotype, and gene expression profile are known to be causally associated with exposure to ionizing radiation (Boerma, Bart & Wondergem, 2002; Salata et al., 2014). This study was conducted to evaluate changes in the gene expression profile of CFs after radiation exposure, particularly the role of miR-21 in this process.

We found that myocardial fibroblasts undergo apoptosis and cycle changes after radiation, the degree of which increase with increasing radiation intensity. In DEG analysis, changes in the gene expression profile of CFs after radiation exposure at low and high doses were detected. Boerma, Bart & Wondergem (2002) demonstrated that genes in CFs are down-regulated more frequently by genechip assay after ionizing radiation, and the degree of changes in gene expression is positively correlated with the radiation dose, which is in agreement with our results. To better understand the relationship between widely altered gene groups, we combined WGCNA and DEG analysis, and found three MEs showing significant changes in expression.

Myocardial fibroblasts were arrested in G2/M phase after irradiation, but the number of cells in G2/M phase decreased significantly after interference with miR-21, suggesting that miR-21 was more responsive to stress after irradiation. This may be a self-protective response of cells by blocking G2/M phase but may also cause cell apoptosis. To specifically identify which genes are affected by miR-21, we searched for Brown module genes, which show lower cell expression after irradiation (possibly because of elevated miR-21). We predicted that among Brown module genes, if genes decreased in the irradiation group compared to in the non-irradiation group are increased after anti-miR-21 treatment, the genes may be affected by miR-21. Through GO and KEGG analysis, we found that the genes are mainly involved in cell differentiation and cycle. The genes involved in G2/M transformation are *Cenpf* and *Cna2*. However, according to an online prediction website, these two genes are not the targets of miR-21. How these genes are related to and function with miR-21 required further analysis.

We also selected *Rsad2* and *Gdf15* (Ek et al., 2016) as candidate genes predicted as target genes of miR-21 by bioinformatic algorithms. Our study suggested that the expression of *Rsad2* and *Gdf15* were up-regulated when miR-21 expression was decreased with an miR-21 inhibitor in 8.5-Gy irradiated cells. The target genes in irradiated CFs may be regulated by various ways, and thus, the down-regulation of target gene expression caused by miR-21 was neutralized via multiple pathways. Therefore, the hub target genes specifically regulated by miR-21 require further experimental analysis and are the focus of our future studies.

Radiation is known to cause fibrosis of the heart. Importantly, we found that some genes in the Yellow or Turquoise gene modules were over-expressed after irradiation, GO analysis showed that these genes were involved in extracellular space and extracellular matrix. We also selected several genes showing obvious differences (—log2—>2, *p* < 0.05) in the sequencing results for Q-PCR verification. These genes were *Grem1*, *Clu*, *Gdf15*, *Ccl7*, and *Cxcl1*, all of which showed increased expression to varying degrees, which is consistent with the sequencing results. Because the extracellular space and extracellular matrix may play an important role in the occurrence of cell fibrosis, we predicted that changes in these genes are related to fibrosis of the heart after radiation. *Grem1* is a candidate gene responsible for *in vitro* cardiomyogenic differentiation (Kami et al., 2008). Mueller et al. (2013) studied 214 patients with non-ischemic heart failure and found that the expression of *Grem1* was significantly associated with the degree of myocardial fibrosis and was an independent predictor of poor prognosis in patients with non-ischemic heart failure. In transgenic mice, *Grem1* was found to regulate anti-fibrotic chemokine production and led to fibrosis (Koli et al., 2016). Clusterin (*Clu*) is a molecular chaperone that protects cellular proteins (Li et al., 2011) and is thought to promote survival by reducing oxidative stress (Park, Mathis & Lee, 2014), and can be induced in myocarditis and numerous inflammatory injuries (McLaughlin et al., 2000). *Clu*-deficient mice exhibited cardiac function impairment and severe myocardial scarring (McLaughlin et al., 2000). Circulating Clu levels are associated with left ventricular remodelling after myocardial infarction (Turkieh et al., 2018). *Clu* also has a potential anti-atherosclerotic effect by inducing cholesterol export from macrophage foam cells upon repeated ischemia/reperfusion injury (Gelissen et al., 1998). Growth differentiation factor 15 (*Gdf15*) is a heart-derived hormone (Wang et al., 2017), also known as *MIC-1*, and is a member of the transforming growth factor-β (*TGF-*β**) superfamily. This protein is thought to be associated with heart failure, all-cause mortality (Wiklund et al., 2010), atherosclerosis, and metabolism (Emmerson et al., 2017). *Gdf15* is considered as a novel antihypertrophic regulatory factor in the heart (Xu et al., 2006), which can elicit SMAD2/3 and then antihypertrophic effects. Additionally, overexpression *Smad7* (target gene of miR-21) reversed the antihypertrophic effects of *Gdf15* (Xu et al., 2006), suggesting that increased miR-21 after irradiation inhibits its target gene *Smad7*, promotes cardiac hypertrophy, and thereby neutralizes the anti-hypertrophic effect of elevated *Gdf15*. It has been shown that local targeting of miR-21 have potential therapeutic utility in mitigating radiation-induced lung fibrosis (Kwon et al., 2016). Therefore, *Gdf15* both as a target of miR-21 and as a fibrosis factor showing its potential ability for treatment of cardiac fibrosis after radiation. Our study showed that *Gdf15* expression was higher after interference with the expression of miR-21 in irradiated CFs, indicating that *Gdf15*-related anti-fibrosis was inhibited by miR-21. As a member of the chemokine family, *Ccl7* plays an important role in cardiac hypertrophy. The expression of chemokine mRNA in the left ventricular hypertrophy model showed that *Ccl7* expression was increased sharply in the early inflammatory phase and returned to baseline in the hypertrophic phase (Nemska et al., 2016). One of the primary functions of *Ccl7* is to mobilize and promote monocyte migration from the bone marrow to inflamed tissue (Tsou et al., 2007). Another study showed that circulating B cells produce the chemokine *Ccl7* in myocardial infarction, which in turn damages the heart (Kim & Luster, 2013; Zouggari et al., 2013), and thus *Ccl7/ Mcp-3* is involved in impairing cardiac function as a virulence factor. *Cxcl1* as potent neutrophil chemoattractant also plays an important role in a CF ischemia-reperfusion model (Lafontant et al., 2006) and contributes to neointima formation in the blood vessel walls (Yu et al., 2016). Therefore, these two chemokines may be virulence factors in cardiac damage. We will further explore the effects of these gene expression changes on the cell phenotype.

## Conclusions

In conclusion, our results indicate that miR-21 may play major role in promoting fibrosis and G2/M blockade by regulating *Grem1*, *Clu*, *Gdf15*, *Ccl7*, *Cxcl1* and *Rsad2* after irradiation. The pathophysiological role of miR-21, and the potential for manipulating miR-21 to achieve therapeutic effects, should be further explored in RIHD (Supplemental Information 4). We only sequenced primary cells in vitro. In our further studies we will perform single-cell sequencing of rat hearts after irradiation, which may provide more detailed information.

##  Supplemental Information

10.7717/peerj.10502/supp-1 Supplemental Information 1 Flow chart of methods used in this study.

10.7717/peerj.10502/supp-2 Supplemental Information 2 Gene number of each module eigengene.

10.7717/peerj.10502/supp-3 Supplemental Information 3 DEG number for each group.

10.7717/peerj.10502/supp-4 Supplemental Information 4 The possible mechanism of RIHD.

10.7717/peerj.10502/supp-5 Supplemental Information 5 Raw data processed.

10.7717/peerj.10502/supp-6 Supplemental Information 6 FPKM data of all samples.

10.7717/peerj.10502/supp-7 Supplemental Information 7 Code.

## Additional Information and Declarations

### Data Availability

The following information was supplied regarding data availability: The raw data are available in the Supplemental Files. Raw RNA-seq data are available at GEO: PRJNA642331.

## References

[ref-1] Adam O, Lohfelm B, Thum T, Gupta SK, Puhl SL, Schafers HJ, Bohm M, Laufs U (2012). Role of miR-21 in the pathogenesis of atrial fibrosis. Basic Research in Cardiology.

[ref-2] Boerma M, Bart CI, Wondergem J (2002). Effects of ionizing radiation on gene expression in cultured rat heart cells. International Journal of Radiation Biology.

[ref-3] Boero IJ, Paravati AJ, Triplett DP, Hwang L, Matsuno RK, Gillespie EF, Yashar CM, Moiseenko V, Einck JP, Mell LK, Parikh SA, Murphy JD (2016). Modern radiation therapy and cardiac outcomes in breast cancer. International Journal of Radiation Oncology, Biology, Physics.

[ref-4] Dennis Jr G, Sherman BT, Hosack DA, Yang J, Gao W, Lane HC, Lempicki RA (2003). DAVID: database for annotation, visualization, and integrated discovery. Genome Biology.

[ref-5] Ek WE, Hedman AK, Enroth S, Morris AP, Lindgren CM, Mahajan A, Gustafsson S, Gyllensten U, Lind L, Johansson A (2016). Genome-wide DNA methylation study identifies genes associated with the cardiovascular biomarker GDF-15. Human Molecular Genetics.

[ref-6] Emmerson PJ, Wang F, Du Y, Liu Q, Pickard RT, Gonciarz MD, Coskun T, Hamang MJ, Sindelar DK, Ballman KK, Foltz LA, Muppidi A, Alsina-Fernandez J, Barnard GC, Tang JX, Liu X, Mao X, Siegel R, Sloan JH, Mitchell PJ, Zhang BB, Gimeno RE, Shan B, Wu X (2017). The metabolic effects of GDF15 are mediated by the orphan receptor GFRAL. Nature Medicine.

[ref-7] Freeman JL, Weber GJ, Peterson SM, Nie LH (2014). Embryonic ionizing radiation exposure results in expression alterations of genes associated with cardiovascular and neurological development, function, and disease and modified cardiovascular function in zebrafish. Frontiers in Genetics.

[ref-8] Gelissen IC, Hochgrebe T, Wilson MR, Easterbrook-Smith SB, Jessup W, Dean RT, Brown AJ (1998). Apolipoprotein J (clusterin) induces cholesterol export from macrophage-foam cells: a potential anti-atherogenic function?. Biochemical Journal.

[ref-9] Hancock SL, Tucker MA, Hoppe RT (1993). Factors affecting late mortality from heart disease after treatment of Hodgkin’s disease. Journal of the American Medical Association.

[ref-10] Huangda W, Sherman BT, Lempicki RA (2009). Systematic and integrative analysis of large gene lists using DAVID bioinformatics resources. Nature Protocols.

[ref-11] Kami D, Shiojima I, Makino H, Matsumoto K, Takahashi Y, Ishii R, Naito AT, Toyoda M, Saito H, Watanabe M, Komuro I, Umezawa A (2008). Gremlin enhances the determined path to cardiomyogenesis. PLOS ONE.

[ref-12] Kim ND, Luster AD (2013). To B or not to B–that is the question for myocardial infarction. Nature Medicine.

[ref-13] Koli K, Sutinen E, Ronty M, Rantakari P, Fortino V, Pulkkinen V, Greco D, Sipila P, Myllarniemi M (2016). Gremlin-1 overexpression in mouse lung reduces silica-induced lymphocyte recruitment - a link to idiopathic pulmonary fibrosis through negative correlation with CXCL10 chemokine. PLOS ONE.

[ref-14] Kura B, Kalocayova B, Devaux Y, Bartekova M (2020). Potential clinical implications of miR-1 and miR-21 in heart disease and cardioprotection. International Journal of Molecular Sciences.

[ref-15] Kwon OS, Kim KT, Lee E, Kim M, Choi SH, Li H, FornaceJr AJ, Cho JH, Lee YS, Lee JS, Lee YJ, Cha HJ (2016). Induction of MiR-21 by stereotactic body radiotherapy contributes to the pulmonary fibrotic response. PLOS ONE.

[ref-16] Lafontant PJ, Burns AR, Donnachie E, Haudek SB, Smith CW, Entman ML (2006). Oncostatin M differentially regulates CXC chemokines in mouse cardiac fibroblasts. American Journal of Physiology. Cell Physiology.

[ref-17] Langfelder P, Horvath S (2008). WGCNA: an R package for weighted correlation network analysis. BMC Bioinformatics.

[ref-18] Li Q, Yao Y, Shi S, Zhou M, Zhou Y, Wang M, Chiu JJ, Huang Z, Zhang W, Liu M, Wang Q, Tu X (2020). Inhibition of miR-21 alleviated cardiac perivascular fibrosis via repressing EndMT in T1DM. Journal of Cellular and Molecular Medicine.

[ref-19] Li S, Guan Q, Chen Z, Gleave ME, Nguan CY, Du C (2011). Reduction of cold ischemia-reperfusion injury by graft-expressing clusterin in heart transplantation. Journal of Heart and Lung Transplantation.

[ref-20] Love MI, Huber W, Anders S (2014). Moderated estimation of fold change and dispersion for RNA-seq data with DESeq2. Genome Biology.

[ref-21] Ma C, Fu Z, Guo H, Wei H, Zhao X, Li Y (2019). The effects of Radix Angelica Sinensis and Radix Hedysari ultrafiltration extract on X-irradiation-induced myocardial fibrosis in rats. Biomedicine and Pharmacotherapy.

[ref-22] Ma CX, Zhao XK, Li YD (2019). New therapeutic insights into radiation-induced myocardial fibrosis. Therapeutic Advances in Chronic Disease.

[ref-23] McLaughlin L, Zhu G, Mistry M, Ley-Ebert C, Stuart WD, Florio CJ, Groen PA, Witt SA, Kimball TR, Witte DP, Harmony JA, Aronow BJ (2000). Apolipoprotein J/clusterin limits the severity of murine autoimmune myocarditis. Journal of Clinical Investigation.

[ref-24] Moore-Morris T, Guimaraes-Camboa N, Banerjee I, Zambon AC, Kisseleva T, Velayoudon A, Stallcup WB, Gu Y, Dalton ND, Cedenilla M, Gomez-Amaro R, Zhou B, Brenner DA, Peterson KL, Chen J, Evans SM (2014). Resident fibroblast lineages mediate pressure overload-induced cardiac fibrosis. Journal of Clinical Investigation.

[ref-25] Mortazavi A, Williams BA, McCue K, Schaeffer L, Wold B (2008). Mapping and quantifying mammalian transcriptomes by RNA-Seq. Nature Methods.

[ref-26] Mueller KA, Tavlaki E, Schneider M, Jorbenadze R, Geisler T, Kandolf R, Gawaz M, Mueller II, Zuern CS (2013). Gremlin-1 identifies fibrosis and predicts adverse outcome in patients with heart failure undergoing endomyocardial biopsy. Journal of Cardiac Failure.

[ref-27] Nemska S, Monassier L, Gassmann M, Frossard N, Tavakoli R (2016). Kinetic mRNA profiling in a rat model of left-ventricular hypertrophy reveals early expression of chemokines and their receptors. PLOS ONE.

[ref-28] Park S, Mathis KW, Lee IK (2014). The physiological roles of apolipoprotein J/clusterin in metabolic and cardiovascular diseases. Reviews in Endocrine and Metabolic Disorders.

[ref-29] Rademaker J, Schoder H, Ariaratnam NS, Strauss HW, Yahalom J, Steingart R, Oeffinger KC (2008). Coronary artery disease after radiation therapy for Hodgkin’s lymphoma: coronary CT angiography findings and calcium scores in nine asymptomatic patients. American Journal of Roentgenology.

[ref-30] Salata C, Ferreira-Machado SC, De Andrade CB, Mencalha AL, Mandarim-De-Lacerda CA, deAlmeida CE (2014). Apoptosis induction of cardiomyocytes and subsequent fibrosis after irradiation and neoadjuvant chemotherapy. International Journal of Radiation Biology.

[ref-31] Szklarczyk D, Gable AL, Lyon D, Junge A, Wyder S, Huerta-Cepas J, Simonovic M, Doncheva NT, Morris JH, Bork P, Jensen LJ, Mering CV (2019). STRING v11: protein-protein association networks with increased coverage, supporting functional discovery in genome-wide experimental datasets. Nucleic Acids Research.

[ref-32] Thum T, Gross C, Fiedler J, Fischer T, Kissler S, Bussen M, Galuppo P, Just S, Rottbauer W, Frantz S, Castoldi M, Soutschek J, Koteliansky V, Rosenwald A, Basson MA, Licht JD, Pena JT, Rouhanifard SH, Muckenthaler MU, Tuschl T, Martin GR, Bauersachs J, Engelhardt S (2008). MicroRNA-21 contributes to myocardial disease by stimulating MAP kinase signalling in fibroblasts. Nature.

[ref-33] Tsou CL, Peters W, Si Y, Slaymaker S, Aslanian AM, Weisberg SP, Mack M, Charo IF (2007). Critical roles for CCR2 and MCP-3 in monocyte mobilization from bone marrow and recruitment to inflammatory sites. Journal of Clinical Investigation.

[ref-34] Turkieh A, Fertin M, Bouvet M, Mulder P, Drobecq H, Lemesle G, Lamblin N, De Groote P, Porouchani S, Chwastyniak M, Beseme O, Amouyel P, Mouquet F, Balligand JL, Richard V, Bauters C, Pinet F (2018). Expression and implication of clusterin in left ventricular remodeling after myocardial infarction. Circulation: Heart Failure.

[ref-35] Van Leeuwen FE, Ng AK (2016). Long-term risk of second malignancy and cardiovascular disease after Hodgkin lymphoma treatment. Hematology, ASH Education Program.

[ref-36] Wang H, Wei J, Zheng Q, Meng L, Xin Y, Yin X, Jiang X (2019). Radiation-induced heart disease: a review of classification, mechanism and prevention. International Journal of Biological Sciences.

[ref-37] Wang T, Liu J, McDonald C, Lupino K, Zhai X, Wilkins BJ, Hakonarson H, Pei L (2017). GDF15 is a heart-derived hormone that regulates body growth. EMBO Molecular Medicine.

[ref-38] Wiklund FE, Bennet AM, Magnusson PK, Eriksson UK, Lindmark F, Wu L, Yaghoutyfam N, Marquis CP, Stattin P, Pedersen NL, Adami HO, Gronberg H, Breit SN, Brown DA (2010). Macrophage inhibitory cytokine-1 (MIC-1/GDF15): a new marker of all-cause mortality. Aging Cell.

[ref-39] Xu J, Kimball TR, Lorenz JN, Brown DA, Bauskin AR, Klevitsky R, Hewett TE, Breit SN, Molkentin JD (2006). GDF15/MIC-1 functions as a protective and antihypertrophic factor released from the myocardium in association with SMAD protein activation. Circulation Research.

[ref-40] Yu B, Wong MM, Potter CM, Simpson RM, Karamariti E, Zhang Z, Zeng L, Warren D, Hu Y, Wang W, Xu Q (2016). Vascular stem/progenitor cell migration induced by smooth muscle cell-derived chemokine (C-C Motif) ligand 2 and chemokine (C-X-C motif) ligand 1 contributes to neointima formation. Stem Cells.

[ref-41] Zhao H, Zhuang Y, Li R, Liu Y, Mei Z, He Z, Zhou F, Zhou Y (2019). Effects of different doses of X-ray irradiation on cell apoptosis, cell cycle, DNA damage repair and glycolysis in HeLa cells. Oncology Letters.

[ref-42] Zhou XL, Xu H, Liu ZB, Wu QC, Zhu RR, Liu JC (2018). miR-21 promotes cardiac fibroblast-to-myofibroblast transformation and myocardial fibrosis by targeting Jagged1. Journal of Cellular and Molecular Medicine.

[ref-43] Zouggari Y, Ait-Oufella H, Bonnin P, Simon T, Sage AP, Guerin C, Vilar J, Caligiuri G, Tsiantoulas D, Laurans L, Dumeau E, Kotti S, Bruneval P, Charo IF, Binder CJ, Danchin N, Tedgui A, Tedder TF, Silvestre JS, Mallat Z (2013). B lymphocytes trigger monocyte mobilization and impair heart function after acute myocardial infarction. Nature Medicine.

