# Supplementary figures and images for: miR-21 is upregulated, promoting fibrosis and blocking G2/M in irradiated rat cardiac fibroblasts
Source: PeerJ. 2020 Dec 10;8:e10502. doi: 10.7717/peerj.10502 (PMC7733651; doi:10.7717/peerj.10502)

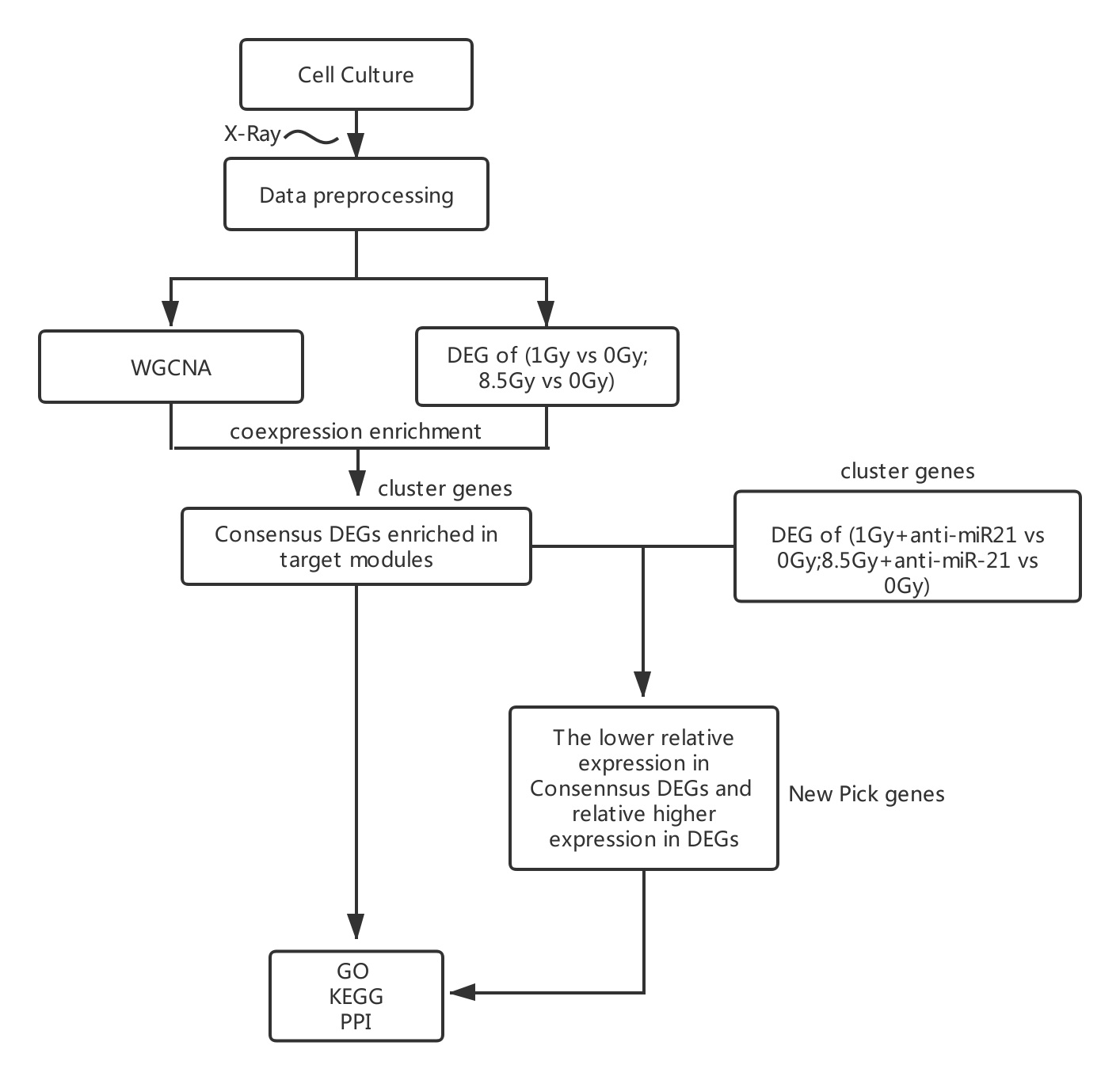

Supplement: Supplemental Information 1 [file peerj-08-10502-s001.png]

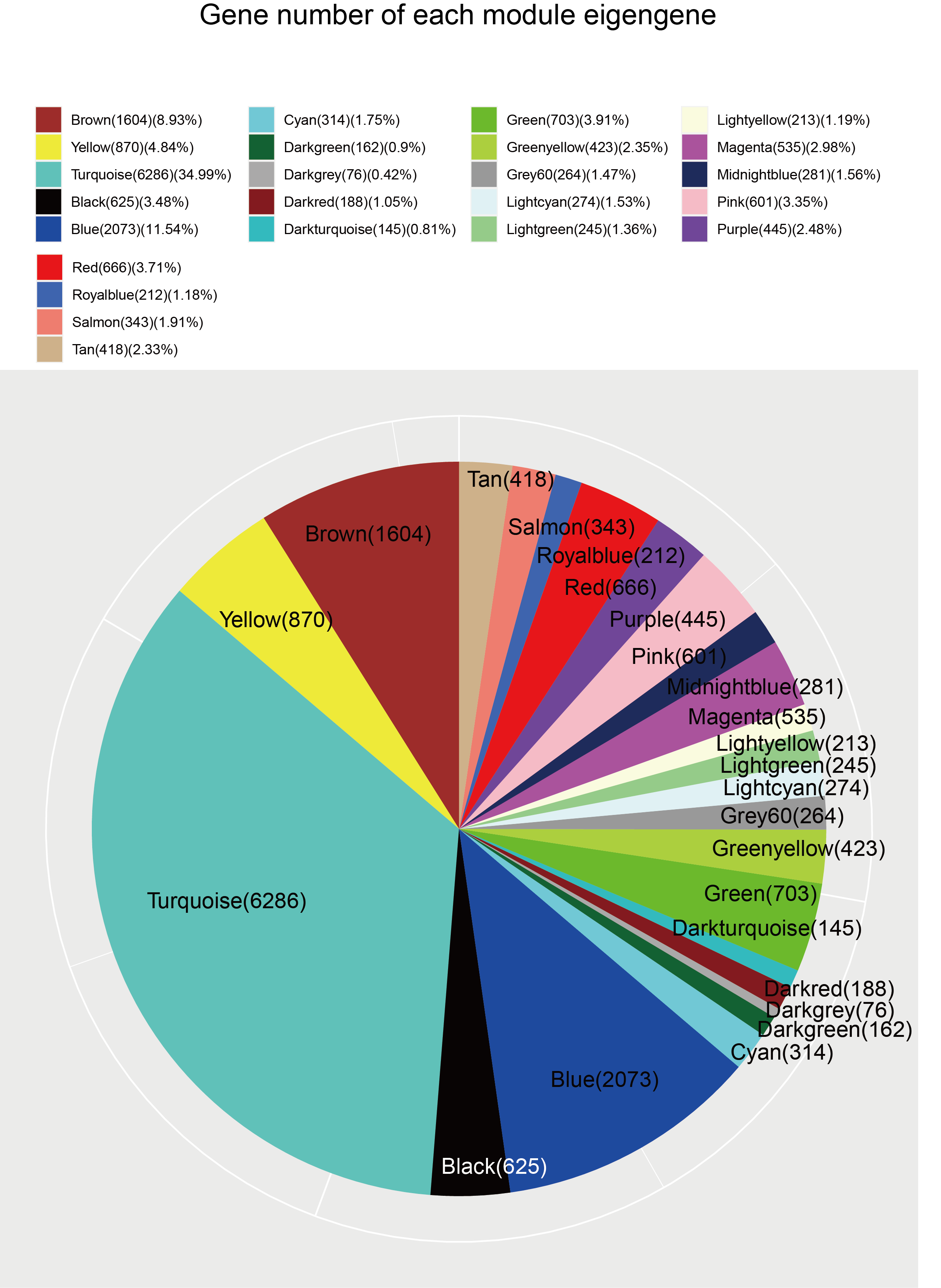

Supplement: Supplemental Information 2 [file peerj-08-10502-s002.png]

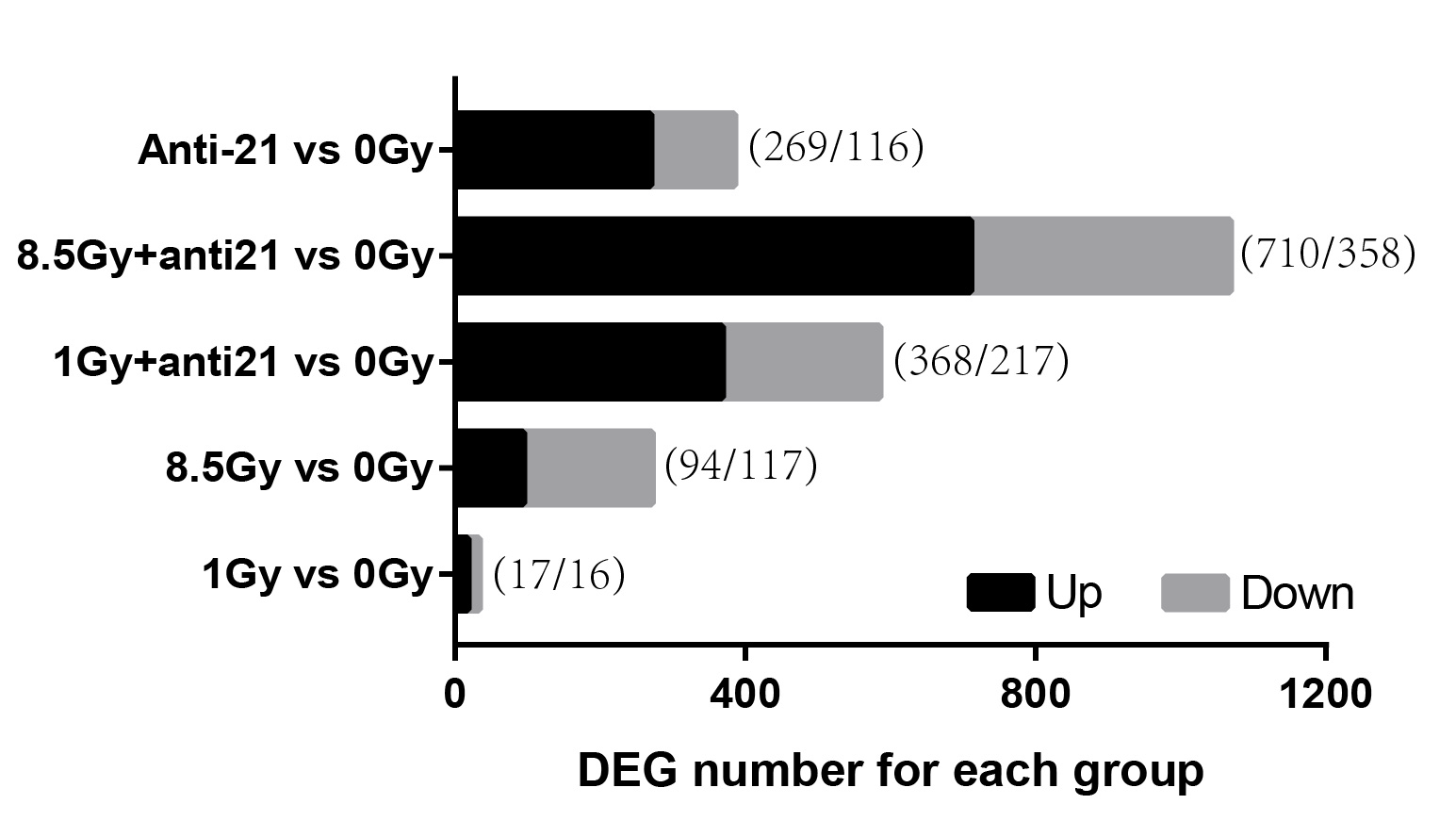

Supplement: Supplemental Information 3 [file peerj-08-10502-s003.jpg]

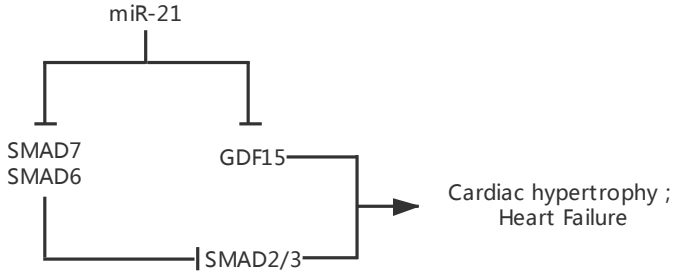

Supplement: Supplemental Information 4 [file peerj-08-10502-s004.pdf]
